# Disclosure of clinically actionable genetic variants to thoracic aortic dissection biobank participants

**DOI:** 10.1186/s12920-021-00902-5

**Published:** 2021-03-01

**Authors:** Adelyn Beil, Whitney Hornsby, Wendy R. Uhlmann, Rajani Aatre, Patricia Arscott, Brooke Wolford, Kim A. Eagle, Bo Yang, Jennifer McNamara, Cristen Willer, J. Scott Roberts

**Affiliations:** 1 Division of Pediatric Genetics, Metabolism, and Genomic Medicine, Department of Pediatrics, Michigan Medicine, Ann Arbor, MI 48109 USA; 2 Department of Internal Medicine, Michigan Medicine, 5804 Medical Science II, 1241 E. Catherine Street, Ann Arbor, MI 48109-5618 USA; 3 Department of Human Genetics, University of Michigan Medical School, Ann Arbor, MI 48109 USA; 4 Center for Bioethics and Social Sciences in Medicine, University of Michigan, Ann Arbor, MI 48109-2029 USA; 5 Department of Computational Medicine and Bioinformatics, University of Michigan Medical School, Ann Arbor, MI 48109 USA; 6 Department of Cardiac Surgery, Michigan Medicine, Ann Arbor, MI 48109 USA; 7 Department of Health Behavior and Health Education, School of Public Health, University of Michigan, Ann Arbor, MI 48109 USA

**Keywords:** Biobank, Communication, Genetic counseling, Pathogenic variants, Return of results

## Abstract

**Background:**

Disclosure of pathogenic variants to thoracic aortic dissection biobank participants was implemented. The impact and costs, including confirmatory genetic testing in a Clinical Laboratory Improvement Amendments (CLIA)-certified laboratory, were evaluated.

**Methods:**

We exome sequenced 240 cases with thoracic aortic dissection and 258 controls, then examined 11 aortopathy genes. Pathogenic variants in 6 aortopathy genes (*COL3A1*, *FBN1*, *LOX*, *PRKG1*, *SMAD3*, and *TGFBR2)* were identified in 26 participants, representing 10.8% of the cohort (26/240). A second research sample was used to validate the initial findings. Mailed letters to participants disclosed that a potentially disease causing DNA alteration had been identified (neither the gene nor variant was disclosed). Participants were offered clinical genetic counseling and confirmatory genetic testing in a CLIA laboratory.

**Results:**

Excluding 6 participants who were deceased or lost to follow-up, 20 participants received the disclosure letter, 10 of whom proceeded with genetic counseling, confirmatory genetic testing, and enrolled in a survey study. Participants reported satisfaction with the letter (4.2 ± 0.7) and genetic counseling (4.4 ± 0.4; [out of 5, respectively]). The psychosocial impact was characterized by low decisional regret (11.5 ± 11.6) and distress (16.0 ± 4.2, [out of 100, respectively]). The average cost for 26 participants was $400, including validation and sending letters. The average cost for those who received genetic counseling and CLIA laboratory confirmation was $605.

**Conclusions:**

Participants were satisfied with the return of clinically significant biobank genetic results and CLIA laboratory testing; however, the process required significant time and resources. These findings illustrate the trade-offs involved for researchers considering returning research genetic results.

**Supplementary Information:**

The online version contains supplementary material available at 10.1186/s12920-021-00902-5.

## Background

Genetic research studies generate individual results that may have clinical implications for participants, but these findings require confirmatory genetic testing in a Clinical Laboratory Improvement Amendments (CLIA) laboratory prior to use in clinical care [1, 2]. The Presidential Commission on the Study of Bioethical Issues recommends that researchers move towards returning genetic results, including clinically significant secondary results [3]. In 2018, the National Academies of Sciences, Engineering, and Medicine advocated for returning research genetic results to benefit participants and advance research. This report highlighted the need to assess participant, physician, and researcher preferences [4] to guide results return processes. In 2019, the American Society of Human Genetics released a position statement on investigators’ responsibility to recontact research participants, especially if a clinically actionable variant is identified [5]. In situations where research genetic results are clinically actionable, researchers may also feel an ethical obligation or simply that it is helpful to the participant to disclose this information [6].

Participants report high levels of interest in receiving research genetic results that are clinically actionable [7–9], defined as a change in how a clinician would manage a patient’s risk of disease relative to the current clinical plan. However, research genetic results are rarely returned to participants in genetic studies, for reasons including: (1) the higher error rate observed in research-grade data relative to clinical-grade genetic data; (2) difficulty interpreting the clinical implications of the genetic information; and (3) lack of expertise and resources to effectively convey results to participants using clinical protocols [10, 11]. There is also limited guidance on how the recontact process should be operationalized, including how (and if) to confirm the genetic variant, what variants to disclose, how to inform participants, who should disclose results, and who should cover the costs [10]. Addressing these unanswered ethical and practical questions is critical to ensure responsible practices when returning research genetic results.

We sought to address these questions among thoracic aortic dissection biobank participants given that between 10 and 25% of patients with a thoracic aortic aneurysm or dissection are estimated to have an underlying genetic predisposition (e.g., Marfan syndrome) [12, 13]. These are conditions with significant morbidity and mortality and routine clinical and surgical management are critical to saving lives. Moreover, knowledge of these results after clinical confirmation support cascade screening for at-risk first-degree family members, whose conditions may otherwise go undetected [14]. Specifically, we developed a process for recontact and disclosure of pathogenetic variants and assessed the impact and associated costs of disclosure and confirmatory genetic testing in a CLIA-approved laboratory.

## Methods

Participants were recruited to the Cardiovascular Health Improvement Project (CHIP), a longitudinal cardiovascular biobank within the Michigan Medicine Frankel Cardiovascular Center, initiated in 2013; biobank recruitment is ongoing [15]. The primary focus is to recruit patients with aortic disease; governance is provided by three executive committees (Additional file 1, Committees). The University of Michigan Institutional Review Board (IRBMED) approved all protocols and procedures (HUM00052866). Eligible participants providing written informed consent between 2012 and 2015 were informed that genetic results may be returned in the future. All aortic cases represented here were enrolled in this manner. In February 2016, the language in the informed consent form was amended and specifically asked participants to opt-in to receive research genetic results for cardiovascular disease and for other diseases (e.g. cancer). After opt-in became available in 2016, 94% of participants (3423/3627) requested to have clinically-actionable variants related to cardiovascular disease returned. For both consent processes described above, participants were informed that the decision to return results would be based on medical expertise and access to sufficient resources (e.g., time and funding). The research was performed in accordance with the Declaration of Helsinki.

### Whole exome sequencing and variant annotation

Whole exome sequencing was performed on a subset of biobank participants, including 240 patients with thoracic aortic dissection or rupture and 258 controls matched for age, sex, and ancestry with no cardiovascular conditions from the Michigan Genomics Initiative. Detailed methods on sequencing, variant calling, and quality control has been previously published [13]. In brief, an external laboratory blinded to case–control status, identified variants in 11 genes (*COL3A1, FBN1, SMAD3, TGFB2, TGFBR1, TGFBR2, ACTA2, MYH11, MYLK, LOX, PRKG1*), which were annotated and categorized as pathogenic, of unknown significance, or benign for aortic dissection according to the American College of Medical Genetics (ACMG) criteria [12, 13, 16]. Findings pertaining to patient demographics, clinical characteristics, risks factors and surgical outcomes have been previously published [13, 17].

### Operational steps for recontact and disclosure

**Fig. 1:**
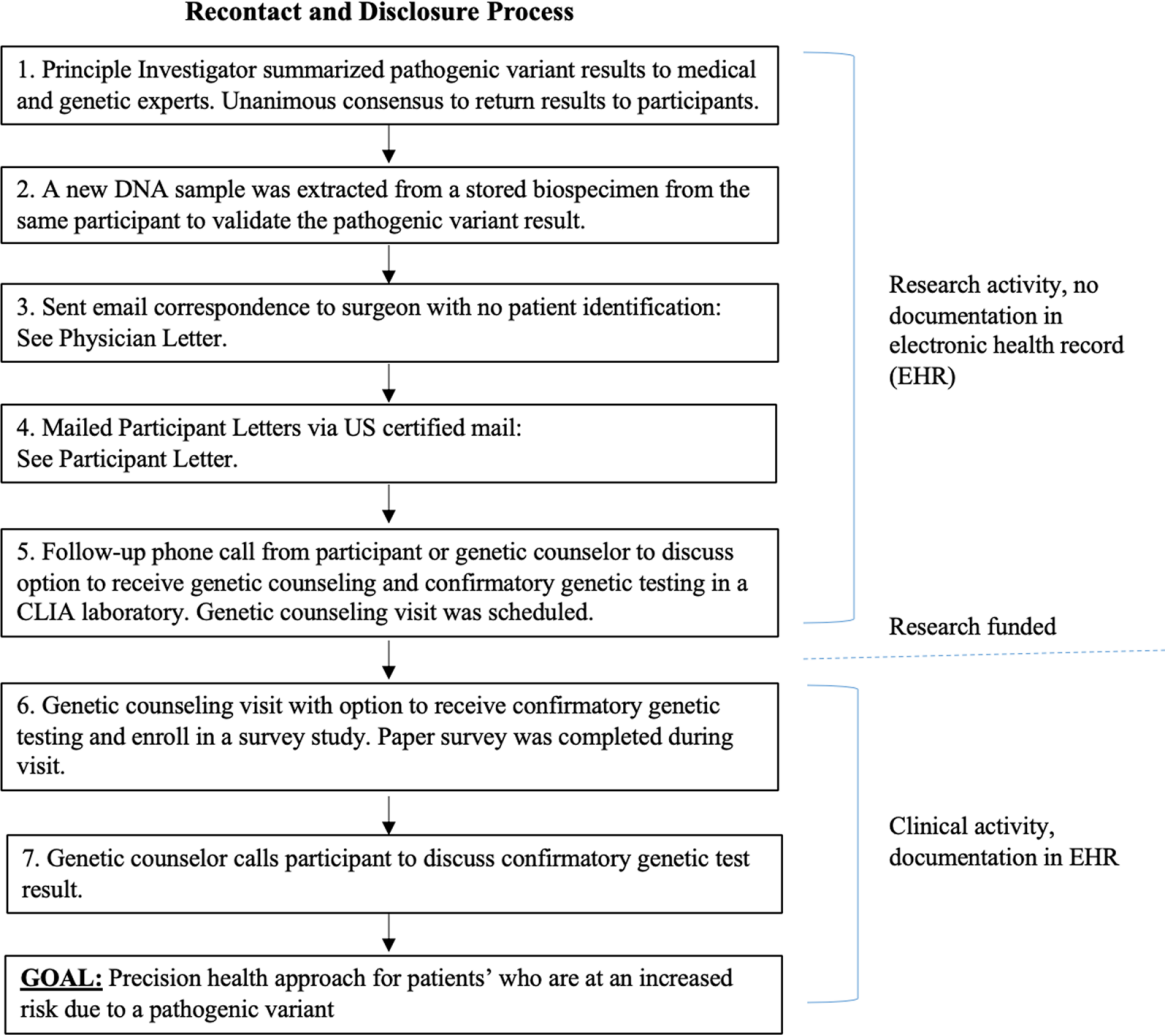
Operational steps implemented for recontact and disclosure of research (non-CLIA) genetic results to thoracic aortic dissection biobank participants

**Fig. 2:**
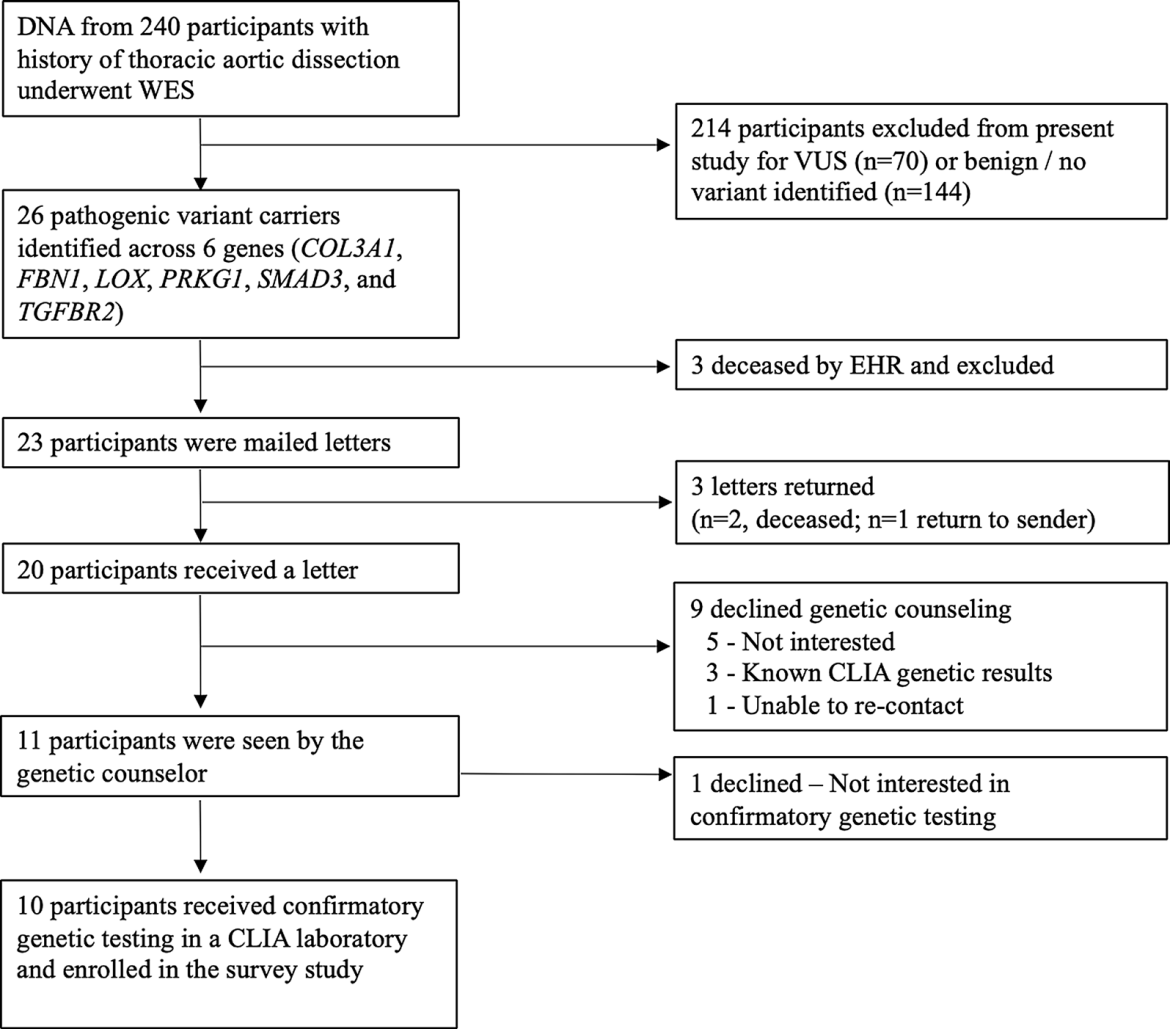
Participant Enrollment. The final cohort consisted of 20 participants, with 10 (50%, 10/20) receiving genetic counseling, confirmatory genetic testing in a CLIA-laboratory, and consenting to a survey study

Additional file 1: Table S1 summarizes how decisions were made and by whom, which led to the operational steps implemented for recontact and disclosure of the pathogenic variants identified in 26 individuals (Figs. 1, 2). In brief, the principal investigator summarized the de-identified pathogenic variant results to medical and genetic experts (e.g., surgeons and the CHIP Medical Findings and Steering Committees, Additional file 1: Material 1). It was unanimously agreed upon that participants should be recontacted as clinical care for themselves and/or at-risk family members (i.e., surveillance, surgical management) would change based on having a pathogenic variant [14]. Next, to confirm the presence of pathogenic variants, a new DNA sample was extracted from a stored biospecimen (a different biospecimen from the same participant), and targeted sequencing using molecular inversion probes (MIPs) methodology was used and 100% pathogenic variant replication was observed [13].

We opted to initiate recontact via US certified mail based on survey results from 250 participants enrolled in CHIP biobank wherein 94% indicated their preference to receive clinically actionable aortic results [18]. This process provided a guaranteed documentation of letter receipt without disclosing the gene or variant to preserve the participant’s “right not to know” and to opt-in: (1) to learn their research genetic result; and (2) to receive genetic counseling, which would be documented into the Electronic Health Record (EHR).

Prior to mailing letters, the cardiothoracic surgeons overseeing the participants’ clinical care were contacted via email and informed that one of their patients (identity was not disclosed) would be receiving a letter indicating that he/she likely carried an alteration in their DNA that may cause aortic disease. The cardiothoracic surgeons were also informed that genetic counseling and confirmatory genetic testing would be offered at no cost. The purpose of the email was to prepare the cardiothoracic surgeons in the event that their patient(s) would ask about the letter and/or their genetic status (Additional file 1: Material 2, Physician Letter). Next, known living participants were mailed letters (neither the gene nor variant was disclosed), which prompted interested recipients to schedule an appointment with a board certified genetic counselor with cardiovascular genetics expertise (Additional file 1: Material 3, Participant Letter).

### Disclosure process

Participants responded to the letter in one of the following ways:

1. They received the letter, made a return phone call to the genetic counselor (or the genetic counselor phoned the participant after a week), and scheduled a genetic counseling appointment with the option to receive confirmatory genetic testing in a CLIA laboratory (paid by the research study). Participants were informed that the clinical genetic counseling visit and confirmatory genetic testing would be documented in EHR. Study participants were informed that the research identified a possible causative variant with the option of CLIA confirmation to validate the finding clinically and make it available for extended family. The benefits and limitations of confirmatory genetic testing were discussed with the participant, as well as the implications of genetic testing and clinical screening for themselves and their families. A detailed 3-generation family history was obtained which could be used to aid potential future cascade testing. Supportive resources both within the healthcare system and through patient support organizations were also discussed with participants. At the end of the genetic counseling visit, participants were invited to participate in a survey study about the recontact and disclosure process. Participant interested in receiving CLIA laboratory genetic testing and participating in the survey study provided separate written informed consent (University of Michigan, IRBMED, HUM00146932).
2. They declined genetic counseling and confirmatory genetic testing in a CLIA laboratory during the phone call with the genetic counselor.
3. They did not respond as they were deceased or lost to follow-up.

### Impact of process

#### Participants’ characteristics, understanding of results, and satisfaction with disclosure process

Demographics and clinical outcomes were collected from EHR. An example of the paper survey is provided (Additional file 1: Material 4). To summarize, socioeconomic characteristics, family health history, and history of genetic counseling and genetic testing were collected via self-report. Participant understanding of their condition, the gene associated with their condition, inheritance pattern, and inheritance risk to siblings and children were assessed via novel questions created by study team members with expertise in genetics, public health, survey development, and cardiogenetics. Participant satisfaction with different elements of the results return process—the letter content, length, comprehensibility, resources provided, and information on family member risk—was assessed using Likert scales (1 = Very Unsatisfied to 5 = Very Satisfied). Participant satisfaction with genetic counseling content and process was assessed by the validated 6-item Genetic Counseling Satisfaction scale (items rated from 1 = Strongly Disagree, to 5 = Strongly Agree) [19]. All survey questions measured at or below the eighth grade level on the Flesch-Kincaid readability scale.

#### Preferences and Information sharing

Six survey items were developed to assess participant preferences regarding mode of results return (multiple-choice), timing of genetic counseling appointment (multiple-choice), and concerns (open-ended). Information sharing was assessed by asking participants to indicate who they informed from a list of family members and other individuals (e.g. physician).

#### Psychological impact and decisional satisfaction and regret

We assessed the psychosocial impact of receiving genetic research results using the 12-item ‘Feelings About genomiC Testing Results’ (FACToR) Scale. The validated scale includes four subscales, with scores ranging from 0–12 for negative emotions, 0–16 for positive emotions, 0–8 for uncertainty, and 0–8 on privacy. An overall score is generated from the subscales, with higher scores being indicative of higher psychological impairment (i.e. stress) [20]. A validated 5-item scale assessed participants’ level of regret regarding their decision to learn their genetic research results. Scores range from 0 to 100, with higher scores indicating higher levels of decisional regret [21].

### Cost analysis

Targeted sequencing using molecular inversion probes (J. Kitzman Laboratory, University of Michigan) was utilized to validate the pathogenic variants identified by the Northwest Genomic Center. Research coordinator time was tracked for letter preparation/mailing, tracking delivery of letter, and project facilitation (hourly rate of $32.21). Genetic counselor time was tracked for all phone calls and face-to-face time with participants (hourly rate of $37.50). The costs for the room charge for genetic counseling visit, phlebotomy and CLIA laboratory genetic testing, in which the gene was sequenced to validate the research genetic result (Invitae Corporation), were extracted from billing records. Mailing costs were tracked for all letters. We evaluated the total cost of the study and average cost per participant. Separate averages were also calculated for those who pursued genetic counseling and confirmatory genetic testing in a CLIA-laboratory and those who did not. The cost of whole exome sequencing and pathogenic variant annotation were not factored into the cost model as these metrics were a part of the parent research study, which preceded the recontact and disclosure process.

### Statistical analysis

Data analysis was restricted solely to descriptive statistics due to the small sample size. Data is presented as mean (± SD) for continuous data, n (%) for categorical data, and range for minimum and maximum response to survey items. When calculating overall scores, missingness was accounted for by averaging by the number of questions answered.

## Results

### Recontact process

Twenty-six participants (26/240) were found to have pathogenic variants with twenty-four different variants in 6 genes (*COL3A1*, *FBN1*, *LOX*, *PRKG1*, *SMAD3*, and *TGFBR2*). Additional information about the classification of these variants, including mutation types, is found in Table 1 of Wolford, Hornsby et al. [13]. Of 26 participants with pathogenic variants, three participants were known from EHR to be deceased prior to mailing letters, and therefore, 23 letters were mailed. Three letters were returned to sender which prompted further EHR review. Of these, two additional participants were found to be deceased based on the National Death Index [22] and one was lost to follow-up. Causes of deaths were due to aortic disease complications (n = 4) or not known (n = 1); the mean age at death was 59 years (SD ± 13). In response to the 20 letters received, three participants called to make a genetic counseling appointment. The genetic counselor made calls to the 17 non-responders. Six (6/17) participants required only one follow-up call, although an average of four follow-up phone calls were needed to recontact the remaining 11 participants (mostly due to missed calls/placing return phone calls). Fifty-five percent (11/20) of those receiving a letter were seen by a genetic counselor, with 10 of the 11 (91%) participants undergoing confirmatory genetic testing in a CLIA laboratory and consenting to the survey study. Reasons for non-consent were lack of interest (n = 6), previously performed CLIA laboratory genetic testing known from EHR review (n = 3), and not able to re-contact (n = 1) (Fig. 2, Study Enrollment).

**Table 1:** Comparison of clinical characteristics, research variant identified, and CLIA laboratory confirmatory genetic test results

Clinical characteristics					Research genetic testing and validation			Genetic counseling and/or CLIA Lab confirmatory genetic testing uptake		Survey study
Documented in EHR prior to research genetic testing					Research result			Clinically validated		
Gender	Dissection age (years)	Family history (1st degree)	Clinical diagnosis (type of dissection and/or genetic condition)	Variant known through clinical testing	Research gene identified	HGVS notation	Research genetic variant validation (MIPs)	Genetic counseling	CLIA lab Variant confirmation (via our study, Invitae)	Enrolled
Male	35–39	Y	Type A	Y	PRKG1	p.Arg177Gln	Y	Y	Y	Y
Female	50–54	Y	Type B	Y	SMAD3	p.Asn218fs	Y	Y	Y	Y
Female	40–44	Y	Type A, MFS^b^	N	FBN1	p.Arg364*	Y	Y	Y	Y
Male	50–54	Y	Type B, MFS^b^	N	FBN1	p.Arg2057*	Y	Y	Y	Y
Female	40–44	Y	Type B, MFS^b^	N	FBN1	c.1148-2A>G	Y	Y	Y	Y
Female	45–49	Y	Type A, MFS^b^	N	FBN1	p.Glu1811Lys	Y	Y	Y	Y
Female	40–44	N	Type A, MFS^b^	N	FBN1	p.Cys1511Arg	Y	Y	Y	Y
Male	50–54	Y	Type A	N	FBN1	p.Asp530Gly	Y	Y	Y	Y
Female	40–44	Y	Type A, Possible CTD	N	COL3A1	p.Gly378Asp^c^	Y	Y	Y	Y
Female	60–64	N	Type B, MFS	N	FBN1	c.443-1G>A	Y	Y	Y	Y
Male	30–34	N	Type A	N	FBN1	p.Asn2624Ser	Y	Y	N	N
Female	30–34	Y	Type A, MFS^b^	Y	FBN1	p.Asp910His^c^	Y	N	N	N
Female	50–54	Y	Type B	Y	SMAD3	p.Asn218fs	Y	N	N	N
Male	20–24	Y	Type A, MFS^b^	N	FBN1	p.Arg2335fs^c^	Y	N	N	N
Male	20–24	N	Type A, MFS^b^	N	FBN1	p.Cys2496Phe	Y	N	N	N
Male	30–34	Y	Type A, MFS^b^	N	FBN1	p.Gly1316fs	Y	N	N	N
Male	15–19	Y	Type A, MFS^b^	N	FBN1	p.Cys1431Arg^c^	Y	N	N	N
Male	50–54	Y	Type A	N	SMAD3	p.Lys116del^c^	Y	N	N	N
Male	25–29	N	Aortic rupture and unknown CTD	N	FBN1	p.Cys2232Tyr	Y	N	N	N
Male	50–54	Y	Type A	N	LOX	p.Cys244fs	Y	N	N	N
Female^d^	45–49	Y	Type B	N	PRKG1	p.Arg177Gln	Y	N	N	N
Female^d^	15–19	Y	Type A, MFS^b^	N	TGFBR2	p.Trp521*	Y	N	N	N
Male^d^	25–29	Y	Type A, MFS^b^	N	FBN1	p.Cys2535Trp	Y	N	N	N
Male^d^	50–54	Y	Type B, MFS^b^	N	FBN1	p.Cys2258Arg	Y	N	N	N
Female^d^	30–34	Y	Type A, MFS^b^	N	FBN1	p.Thr564fs	Y	N	N	N
Female^d^	20–24	N	Type A, MFS^b^	Y	FBN1	p.Gly1022*	Y	N	N	N

*CLIA* Clinical Laboratory Improvement Amendments, *CTD* connective tissue disease, *MFS* Marfan Syndrome, *MIPs* Molecular Inversion Probe Sequencing, *N* No, *Type A* Type A aortic dissection, *Type B* Type B aortic dissection, *Y* Yes

^a^HGVS mutation (*) means deletion

^b^Participants had a syndromic diagnosis of Marfan Syndrome based on Ghent nosology criteria [28] and the research genetic result aligned with the clinical diagnosis

^c^Genetic variant not present in gnomAD version 2.1, dbSNP version 151, or ClinVar as of September 30, 2018

^d^Deceased (n = 5) or lost to follow-up (n = 1)

### Study timeline

The average time between mailing letters and the genetic counseling visit was 22 ± 19 weeks. The majority of participants did not reside locally and preferred to schedule the genetic counseling visit to coincide with their cardiac surgery return visit. Genetic counselor availability was also a factor although research blocks were created to facilitate participant scheduling. The average time between genetic counseling visit and notification of the confirmatory genetic test result was 4 weeks.

### Participant characteristics

Table 1 provides an overview of clinical characteristics, family history, and the research genetic testing results for all 26 patients. Shaded data is presented (as yes or no) for participants who underwent genetic counseling and confirmatory genetic testing in a CLIA laboratory as well as provided consent to the survey study. The survey cohort (N = 10, percentages below are based on 10 participants) was predominantly female (70%), white (90%), and non-Hispanic (100%); 20% worked fulltime and 30% had earned a college degree. More participants experienced an acute type A aortic dissection (60%) than type B aortic dissection (40%). The mean age at the time of consent was 55 ± 8 years compared to a mean age of 47 ± 8 years at the time of dissection. Additional disease related outcomes are presented in Additional file 1: Table S2. For participants who declined the survey study (N = 10), mean age at the time of contact was 45 ± 16 years compared to a mean age of 34 ± 14 years at the time of dissection. 80% of the survey cohort had a known family history of aortic disease in at least one first degree family member (compared to 60% for those declining), with 50% of participants having more than one affected family member (compared to 40% for those declining). 90% of the survey cohort had siblings of whom 67% of those siblings were affected. Likewise, 90% of the survey cohort had children of whom 33% of those children were affected.

### Impact of recontact and disclosure

The mean score of comprehension of results was 82% correct ± 26% (interquartile range 80%, 95%). The vast majority of survey respondents were able to correctly identify the name of their condition (80%), gene involved (90%), risk to siblings and children of inheriting this variant (both 90%), and inheritance pattern (60%; Table 2).

**Table 2:** Assessing the impact of recontact and disclosure (n = 10 participants)

Per-person comprehension of results^a^ (% answered correctly)	82% (26%)	20–100%
Name of participant’s condition	8 (80%)	–
Name of gene associated with condition	9 (90%)	–
Type of inheritance pattern	6 (60%)	–
Inheritance risk to biological siblings	9 (90%)	–
Inheritance risk to children	9 (90%)	–
Letter satisfaction^b^	4.2 (0.7)	3.0–5.0
Information about research pathogenic variant	4.1 (0.8)	3.0–5.0
Family member implications	4.4 (0.5)	4.0–5.0
Resources provided	4.1 (0.8)	3.0–5.0
Letter length	4.2 (0.7)	3.0–5.0
Readability of letter	4.1 (0.8)	3.0–5.0
Genetic counseling satisfaction^b^	4.4 (0.4)	3.3–5.0
Empathy demonstrated	4.7 (0.7)	3.0–5.0
Facilitated the decision-making process	4.7 (0.5)	4.0–5.0
Reassured	3.7 (0.8)	2.0–5.0
Appointment duration	4.0 (0.7)	3.0–5.0
Concern demonstrated	4.7 (0.5)	4.0–5.0
Appointment was valuable	4.5 (0.7)	3.0–5.0
Psychological response (FACToR score)		
Psychological distress^c^	16.0 (4.2)	7.0–21.0
Negative feelings	3.7 ± 3.4	0.0–12.0
Uncertainty	2.0 ± 1.7	0.0–5.0
Privacy concerns	1.7 ± 2.0	0.0–5.0
Positive feelings	8.7 ± 3.8	0.0–12.0
Decisional satisfaction and regret		
Regret^c^	11.5 (11.6)	0.0–25.0
Information sharing^d^	9 (90%)	–
Spouse or partner	4 (40%)	–
Children	4 (40%)	–
Siblings	4 (40%)	–
Physician/cardiologist	3 (30%)	–
Parents	2 (20%)	–
Other (i.e., relatives, friends, etc.)	3 (30%)	–

Data Presented as mean (SD) for continuous data, n (%) for categorical data, and range

*FACToR Scale* Feelings About genomiC Testing Result

^a^Indicates the percent answered correctly for the 5 comprehension questions (total 41, out of 50)

^b^Measured on a scaled from 0 to 5 with 5 being very satisfied or strongly agree

^c^Measured on a scale from 0 to 100 with 100 being high psychological distress or high decisional regret

^d^Participants were allowed to select more than one answer for Information Sharing

Participants were satisfied with receiving this information via letter (mean score for overall letter satisfaction was 4.2 ± 0.7; range 3.0–5.0, out of 5), and similar scores were observed for length of letter, readability, resources provided, information about their genetic results, and potential implications for family members (mean scores ranged from 4.1 to 4.4; Table 2). Nine out of ten noted that a letter was their preferred way of receiving this information. In free text provided, two participants indicated their preferences to receive information about the gene itself rather than the letter’s statement “you likely carry an alteration in your DNA that may cause disease”. In reference to this statement specifically, one survey participant shared her concern and worry following receiving the letter, noting that for someone with an aortic condition, this was very “scary” information to receive.

Participants were satisfied with their genetic counseling appointment to discuss the research finding and confirmatory genetic testing (mean 4.4 ± 0.4; range 3.3–5.0, out of 5). Mean satisfaction scores were consistent with the various aspects of the genetic counseling appointment including: appointment duration (4.0 ± 0.7; range 3–5), concern demonstrated (4.7 ± 0.5; range 4–5), empathy demonstrated (4.7 ± 0.7; range 3–5), facilitation of decision-making (4.7 ± 0.5; range 4–5), and the appointment was valuable (4.5 ± 0.7; range 3–5). The lowest mean item pertained to assessing the extent to which respondents felt reassured about their research genetic result (3.5 ± 0.8; range 2–5).

#### Psychological response to results return

We assessed the psychological response of receiving a genetic result using the FACToR Scale. Mean scores for psychological distress immediately after the genetic counseling appointment were low (mean FACToR score was 16.0 ± 4.2, out of 100). Mean subscale scores were low for negative feelings (3.7 ± 3.4; range 0–12, out of 12), uncertainty (2.0 ± 1.7; range: 0–5, out of 8), and privacy concerns (1.7 ± 2.0, range 0–5, out of 8). Positive feelings were near the midpoint, indicating a moderate level of positive emotional responses to the genetic test result (8.7 ± 3.8; range 0–12, out of 16).

Researchers use the term decisional regret to describe distress or remorse as a result of making a specific decision. The mean decisional regret score was 11.5 ± 11.6 (range 0–25, out of 100). These scores indicate low levels of regret about deciding to learn their genetic test results. 90% of participants indicated that they would share this information with someone, and 60% indicated their intention to share the result with first-degree family members (Table 3).

**Table 3:** Average time and costs for recontact and disclosure

	Time (h)	Unit or hourly cost	Total cost	Cost per participant
Research genetic variant validation (n = 26)				
Sample preparation	5.5	$22	$123	$5
DNA retrieval, extraction, aliquoting, and shipping	–	$872	$872	$34
Laboratory technician	24	$13	$310	$12
Variant sequencing (reagents, library preparation)	–	$2,325	$2325	$89
DNA sequencing	–	$694	$694	$27
Subtotal			$4324	$166
Research coordinator (n = 23)				
Study implementation	73.6	$32	$2370	$103
Subtotal	–	–	$2370	$103
Genetic counselor				
Phone calls after letters were sent (n = 23)	4.4	$37.50	$165	$7
Genetic counseling (n = 11)	8.8	$37.50	$328	$ 30
Phone calls for CLIA results disclosure (n = 10)	1.5	$37.50	$56	$6
Subtotal			$548	–
Confirmatory genetic testing				
CLIA Laboratory (n = 10)	–	$2500	$2500	$250
Subtotal			$2500	$250
Ancillary costs				
Mailing costs (n = 23)	–	$210	$210	$9
Research visit room charge (n = 11)	–	$253	$253	$23
Phlebotomy blood draw (n = 10)	–	$200	$200	$20
Subtotal			$663	–
Project total			$10,405	–
Average cost per participant			–	$400

Cost noted above were based on the number of samples or participants (denoted in table by n =) receiving time, confirmatory genetic testing, or other resources (e.g., room visit, phlebotomy blood draw). All subtotals were added to calculate project total; the average cost per participant was calculated by dividing the total cost by 26 participants

### Cost analysis

We carefully enumerated the costs involved in each step of the process: (1) the cost to perform research genetic variant validation on a new DNA sample using MIPs was $4324, (2) the cost for the research coordinator to draft letters and execute study was $2370, (3) the cost for the genetic counselor which included phone calls after the letter was sent to participants, genetic counseling visit, and phone calls for results disclosure after CLIA laboratory genetic testing was $548, (4) the cost for CLIA-certified genetic testing was $2500, and (5) the cost for mailing, room charge, and phlebotomy was $663. The total cost of the process was $10,405. Table 3 provides an itemized account for each cost category outlined above. The average cost per participant was $400 for all 26 participants, although the range was $266 to $605 per participant depending on whether the participant opted-in to genetic counseling and CLIA-laboratory confirmatory genetic testing.

## Discussion

This is the first study to examine the impact and cost of returning pathogenic variants to thoracic aortic dissection biobank participants. Fifty-five percent (11/20) of pathogenic variant carriers attended genetic counseling, with 91% of those (10/11) undergoing CLIA-laboratory confirmatory genetic testing. Participants were satisfied with the disclosure process, generally understood the meaning and implications of test results, and did not experience adverse psychological effects. Through the process described here, clinically significant genetic test results from a research study were conveyed, although the process entailed time, resources, and financial costs that were beyond the budget of the parent study.

The key steps involved in the recontact and disclosure process included: (1) research sample validation; (2) recontact via US certified mail disclosing a DNA alteration that is potentially causing disease; (3) genetic counselor telephoned participants; (4) genetic counseling appointment; and (5) CLIA laboratory confirmatory genetic testing. Papaz et al. [23] implemented a similar process among pediatric cardiovascular biobank participants (e.g., returning only actionable results, validating research samples prior to recontact). Our findings were comparable in regard to the uptake of genetic counseling (60%, 12/20 vs 55%, 11/20 reported here) and confirmatory genetic testing (100% 12/12 vs 91%, 10/11 reported here). CLIA laboratory variant confirmation facilitates more precise care for the patient and cascade screening for known familial pathogenic variants. The latter is significantly less costly and more informative than having at-risk relatives undergo full gene sequencing based on their family history (in the absence of testing an affected family member) or screening echocardiograms for surveillance.

Another key aspect of our study included evaluating the impact of receiving research genetic results. Our findings suggest that participants were satisfied with the process and that they generally understood the meaning and implications of test results. Participants reported high levels of satisfaction with recontact via letter and genetic counseling. Participants reported low test-related distress and minimal regret about their decision to learn their research genetic result, which is consistent with the established literature on the psychological impact of receiving genetic test results [24]. The study team did not include information about the gene or genetic variant in the recontact letter to preserve the participant’s right to choose whether they wanted to learn their genetic result, which is consistent with the approach of other groups [24–27]. However, the ambiguity of a letter with no gene described may lead to higher initial levels of anxiety for a small subset of participants.

Research funds are typically used to support the roll out of returning genetic results to participants. We systematically analyzed the financial costs of the recontact and disclosure process to evaluate the financial undertaking. The total cost was $10,405 for all 26 participants, with an average cost of $400 per participant. The average cost of those choosing to opt-in to genetic counseling and confirmatory genetic testing was $605. Other studies, including Papaz et al. [23] and Christensen et al. [9], reported an average cost of $750 and $679 per participant, respectively, to return clinically actionable research genetic results. This level of additional cost might lead some investigators to conclude that such an approach is not feasible. Given this reality, we recommend building these costs into the study budget on the front-end when applying for grants. Institutional support for return of genomic results would also help lessen the financial burden on individual investigator teams. Investigators may be motivated to return research genetic results to help participants and at-risk family members. Of the participants enrolled in the survey study, 90% indicated that they would share this information with someone, with 60% saying they would share the result with first-degree family members in particular. In some of the family members of pathogenic variant carriers, deaths in the 4th and 5th decade of life had been noted as ‘heart disease’ but may not have been conveyed to the physician or recognized as a potential family history of aortic dissection. In these cases, identification of a familial pathogenic variant inherited within the family might identify additional genetic carriers at risk of a catastrophic aortic dissection and indicate surgery at a lower aortic diameter threshold.

There are a number of study limitations, with the most notable being the low number of participants; however, thoracic aortic dissection is a rare medical complication. Nonetheless, the sample size and disease-specificity constrained the ability to examine group differences in key study outcomes and may limit the generalizability of study findings. We report that 10.8% of our TAAD cases harbored a pathogenic variant [13] which is lower than previous reports [12]. We expect that either the expectation of 25% of cases harboring a pathogenic variant is an overestimate, or that additional variants in this population will be identified by sequencing additional genes, future understanding of variants of unknown significance in these genes, or evaluation of polygenic risks. Additionally, the impact and costs were assessed short-term, and thus we are not able to accurately capture the long-term impact of the process on participants, their families, and the healthcare system.

## Conclusion

As the number of individuals who have genetic testing done on a research basis increases, the need for a proven delivery model of health-related genetic results also increases. This study demonstrates a process by which participants of a thoracic aortic dissection biobank receive results that may change their medical management with low psychological distress and high levels of understanding. The costs, time, and resources involved in disclosure of results were significant; however, knowing these ahead of time allows for planning for results disclosure during study development. Continued consideration for results disclosure following studies completing genetic testing is vital, and these results suggest a process acceptable to both researchers and participants.

## Supplementary Information

**Additional file 1.** See Supplemental Materials for details regarding (1) Table S1. Steps taken that preceded the recontact and disclosure of pathogenic variants, (2) Material 1—Cardiovascular Health Improvement Project Committees, (3) Material 2—Physician Letter, (4) Material 3—Participant Letter, and (5) Material 4—Return of Research-Level Genetic Testing Results Study.

## Data Availability

The datasets generated and/or analyzed during the current study are not available due to privacy that may be compromised (individual level data). Contact the co-corresponding author, Dr. Cristen Willer to request permission to obtain access to the raw data from the Cardiovascular Health Improvement Project, a longitudinal cardiovascular biobank within the Michigan Medicine, Frankel Cardiovascular Center. *Web resources* (Michigan Genomics Initiative, https://www.michigangenomics.org). (Cardiovascular Health Improvement Project, https://www.umcvc.org/cardiovascular-health-improvement-project-chip-study).

## Abbreviations

CLIA: Clinical Laboratory Improvement Amendments
CHIP: Cardiovascular Health Improvement Project
ACMG: American College of Medical Genetics
HER: Electronic Health Record
FACToR: ‘Feelings About genomiC Testing Results’ Scale
SD: Standard deviation
