# Supplementary material for: Disclosure of clinically actionable genetic variants to thoracic aortic dissection biobank participants
Source: BMC Med Genomics. 2021 Mar 1;14:66. doi: 10.1186/s12920-021-00902-5 (PMC7923508; doi:10.1186/s12920-021-00902-5)
Supplement: Supplementary file 1 — Additional file 1. See Supplemental Materials for details regarding (1) Table S1. Steps taken that preceded the recontact and disclosure of pathogenic variants, (2) Material 1—Cardiovascular Health Improvement Project Committees, (3) Material 2—Physician Letter, (4) Material 3—Participant Letter, and (5) Material 4—Return of Research-Level Genetic Testing Results Study. [file 12920_2021_902_MOESM1_ESM.docx]

**Additional file**

| **Table S1. Steps taken that preceded the recontact and disclosure of pathogenic variants** | | | |
| --- | --- | --- | --- |
| Groups | Considerations | Consensus | Implementation |
| Principal Investigator (PI) | PI acknowledged an ethical responsibility to offer participants an option to receive results. |  | PI initiated communication with medical and genetic experts (e.g., surgeons and the CHIP Medical Findings and Steering Committees). It was unanimously agreed upon that participants should be recontacted as clinical care would change. |
| Medical Findings Committee outlined a process for recontact |  |  | Steps 1-6 were presented to the Steering Committee |
|  | 1. How were variants annotated? | 1. Renowned expert in aortic disease variant annotation |  |
|  | 1. What variants to disclose? | 1. Clinically actionable variants only |  |
|  | 1. Should variant validation be obtained? | 1. Variant validation should be performed |  |
|  | 1. How to recontact? | 1. Recontact via letter from PI, with 1-2 F/U phone calls from genetic counselor. |  |
|  | 1. Who should disclose result? | 1. Certified genetic counselor |  |
|  | 1. Who should pay? | 1. Research study provides option to receive genetic counseling and confirmatory genetic testing in CLIA laboratory at no cost |  |
| Steering Committee | - Verify steps and funding | - Steps 1 – 6 and funding approved | A letter without the gene or variant disclosed preserves the right of the participant to choose whether or not they would like to learn their results. |
|  | - Should the impact of the process be evaluated? | - Yes |  |
|  | - Considerations for deceased participants | - Known deceased participants would not be mailed a letter. |  |
|  | - Should the cardiothoracic surgeon be informed of the result? - What information should be documented in EHR? - Should the letter include the variant or disclose that a DNA alteration had been identified, potentially causing disease. | - Yes, but patient identify could not be disclosed with consent (per IRB). - Clinical events including genetic counseling and confirmatory genetic testing in a CLIA laboratory. - Sought guidance on letter content from an ethicist. |  |
| Internal Review Board | Amendment Submission (HUM00052866); New IRB application to evaluate the recontact and disclosure process (HUM00146932). | IRB approval | Study Implemented |

**Material 1.**

**Cardiovascular Health Improvement Project Committees**

**1. Scientific/Steering Committee**

Members of the scientific/steering committee are responsible for the overall direction of the biobank including prospective collection of samples (biological and health data) and the scope of the disease population. Members of this committee: aortic and vascular surgeons, Directors of the Frankel Cardiovascular Center, cardiologists, interventional radiologist, geneticists

**2. Access Committee – Review Proposal / Use of Sample**

Members of the access committee review the scientific merit of potential investigators interested in using the biobank’s samples for potential research. Members of this committee: cardiologists, interventional radiologist, geneticists, basic and translational scientists

**3. Medical Findings Committee / Patient Reporting Flow**

Members of the medical findings committee determine the optimal workflow, given current medical and ethics literature, to report significant results to patients that might improve or impact their medical care. The committee considers whether to pursue CLIA testing of research samples and discusses ways in which these results can be returned with providers and other avenues for return. Members of this committee: Geneticists, genetic counselors, health behavioralist, ethicist, pediatric geneticist, pediatric cardiologists

**Material 2.**

**Physician Letter**

Good Afternoon,

Many of you know that the Cardiovascular Health Improvement Project (CHIP) biobank has completed exome sequencing on 240 thoracic aortic dissection cases with funds from Dr. Cristen Willer through NHLBI funding. These samples were processed at the University of Washington and we have identified 26 research subjects with a pathogenic variant in 1 of the following genes: *FBNl, TGFBR2, COL3Al, SMAD3, LOX and PRKGl*. None of the control samples were found to have a pathogenic variant. In addition, we verified our results through in house sequencing.

Of the 26 participants:

- 7 have had genetic testing and the results match our research level results
- 10 have not had genetic testing but have a clinical diagnosis that matches our research level results
- 5 have not had genetic testing and have no clinical diagnosis to match our research level results
- 1 has not had genetic testing and has a clinical diagnosis that does NOT match our research level results
- 3 are deceased and we will not be returning results at this time to families until further discussion takes place around the best way to do this

At this time, we are using a return of results flow that has been **carefully** crafted after meeting with our Medical Findings Committee and been approved by our Steering Committee.

We are notifying you because one (or more) of the 26 research participants that we are returning results to has been seen by you in regards to their dissection. We will be mailing each research participant a letter (attached) over the next month asking them to contact Rajani Aatre, FCVC Certified Genetic Counselor, in regards to a follow up for a genetic counseling visit. A follow up phone call to the letter will also be made by Rajani. The genetic counseling will be paid for by the research study along with the genetic testing, should they opt in for that.

Although **we are not IRB approved to release results directly to you without patient consent, we wanted you to be aware in case a patient calls you directly and asks for guidance.** If a patient meets with the Genetic Counselor, the results will then be put into the patients' medical record at that time. Should you have any questions or concerns, please feel free to contact myself, Dr. Cristen Willer or Rajani Aatre.

Best Regards,

**Material 3.**

**Participant Letter**

**Michigan Medicine**

Division of Cardiovascular Medicine

Department of Internal Medicine

Frankel Cardiovascular Center Room 2336

1500 East Medical Center Drive

Ann Arbor, MI 48109-5852

**Cristen J. Willer, PhD**

*Associate Professor*

*Department of Internal Medicine*

*Department of Human Genetics*

*Department of Computational Medicine and Bioinformatics*

<<Date>>

«First_Name»«Last_Name»

«Address_Line_1»

«Address_Line_2»

«City», «State» «ZIP_Code»

Dear «First_Name» «Last_Name»,

This letter is in reference to your participation in the “Cardiovascular Health Improvement Project” (CHIP – HUM00052866) research study at the University of Michigan Frankel Cardiovascular Center. When you participated in our research study you provided a blood sample for genetic research. We informed you that we might notify you if we found any DNA variants that would likely impact your medical care.

As you may know, DNA is the genetic make-up you receive from your mother and your father. The purpose of this letter is to inform you **that you likely carry an alteration in your DNA that may cause disease.**  This alteration may cause disease in your aorta (the large blood vessel that comes from your heart). This alteration in your genes was discovered in the research blood sample we obtained from you.

Individuals with genetic variants such as the one found in your research blood sample typically inherit aortic disease in a dominant pattern. This means that each child of a person with a disease-causing variant has a 50% chance of inheriting the variant and developing the disorder. At-risk family members (parents, children, siblings) should consider genetic counseling and testing for the known familial variant. Genetic testing can provide more information to family members and early management can help prevent complications of aortic disease.

Please note that your results were obtained at our research laboratory. A research laboratory does not meet the same federal operating standards as a clinical genetic testing laboratory. Therefore, we recommend making an appointment with a genetic counselor to obtain CLIA-certified genetic results and discuss the clinical implications of your result. After confirming the genetic variant, these results can be used to make medical decisions for your medical management and for testing of your biological family members.

Please contact us at your earliest convenience so we may provide genetic counseling and clinical confirmation of your research result at **no cost to you**. You may contact the Frankel Cardiovascular Call Center at any time to schedule an appointment with a Certified Genetic Counselor to discuss next steps at **888-287-1082**.

We hope this letter provides helpful information regarding your research results and recommendations for following up on these results. Thank you again for your continued participation in our research. Your participation is helping us understand the genetic causes of aortic disease.

Sincerely,

___________________________________ ___________________________________

Cristen Willer, PhD Rajani Aatre, MS, CGC

(Principal Investigator) (Certified Genetic Counselor)

Phone: (855) 345-5162 Phone: (855) 345- 5162

Email: [CHIPresearch@med.umich.edu](mailto:CHIPresearch@med.umich.edu)

**Material 4.**

CHIP ID: ____________________ Date: _____________________

Administration Notes: __________________________________________________________________­­­­­__

__________________________________________________________________________________________________________________________________________________________________________

_____________________________________________________________________________________

Return of Research-Level Genetic Testing Results Study

The main purpose of this study is to examine the process and impact of returning research-level genetic testing results to research participants.

We expect the survey to take around 20-25 minutes of your time.

Please note that you do not need to answer any questions that you feel you do not want to.

**A. Questions about your genetic condition**

We are interested in the cardiac genetic condition you discussed with your genetic counselor today, and what impact that condition may have on your health. Please answer the following questions with your understanding of what risks this cardiac genetic condition may have for your or your family’s health.

***Directions:*** *For multiple choice questions, please circle the letter corresponding to your answer. For fill in the blank questions, please write your answer in the space provided.*

1. What is the name of the condition that you discussed today with your genetic counselor?

­____________________________________________________________________/ I do not remember

2. What is the name of the gene that is associated with this condition?

____________________________________________________________________/ I do not remember

3. How long have you been diagnosed with the condition that you discussed with your genetic counselor? *(Please indicate number of years or months, or check new diagnosis)*

____years *or* if less than 1 year, ____months *or*  ____new diagnosis

4. You may have talked with your genetic counselor about how this condition runs through families. Which of the following patterns of inheritance does your condition follow?

a. Autosomal recessive

b. Autosomal dominant

c. X-linked recessive

d. X-linked dominant

e. Multifactorial

f. Other: ­_________________________

g. I do not remember

h. Did not discuss

5. For people with the same condition that you have, what is the chance that their children will also inherit the gene change that can cause this condition?

a. 15%

b. 25%

c. 33%

d. 50%

e. 66%

f. 75%

g. Other: _________

h. I do not remember

6. For people with the same condition that you have, what is the chance that their siblings will also inherit the gene change that can cause this condition?

a. 15%

b. 25%

c. 33%

d. 50%

e. 66%

f. 75%

g. Other: _________

h. I do not remember

**B. Questions about the letter you received about your research-level genetic test results**

We are interested in your thoughts on the letter that you received from the CHIP research study about your research-level genetic test results.

***Directions:*** *Please indicate your degree of satisfaction with the following aspects of the letter you received in the mail about your research-level genetic testing results.*

6. Did you share the information in the letter with anyone? If yes, check all that apply.

__Spouse/partner __Child(ren) __Parents __Sibling(s) __Other Relatives

__Primary care doctor __Cardiologist __Other healthcare provider __Friends

__Other: _______________________________

7. After receiving the letter, did you look online for additional information?

__Yes __No

7a. If yes, what information were you seeking: ____________________________________________

______________________________________________________________________________

______________________________________________________________________________

7b. Were there websites that you found helpful/that you would recommend? *(Please share below)*

____________________________________________________________________________________________________________________________________________________________

______________________________________________________________________________

8. Would you have preferred another way to first receive information on your research-level genetic test results, or is a letter your preferred way?

a. The letter is my preferred way

b. I would have preferred a different way

8a. If you answered letter ‘b’ for the above question, which of the following methods would you have most preferred to first receive information on your research-level genetic test results?

a. Password protected patient portal

b. Email

c. Phone call

d. In-person appointment

e. Other: _______________________________________________________________________

9. Do you still have the letter that was sent to you in the mail? __Yes __No

10. Did you share the letter with anyone? __Yes __No

If yes, please indicate who below:

__Spouse/partner __Child(ren) __Parents __Sibling(s) __Other Relatives

__Primary care doctor __Cardiologist __Other healthcare provider __Friends

__Other: _______________________________

11. Was there content that you did not understand or that was confusing to you? __Yes __No

10a. If yes, what content was confusing or hard to understand? *(Please explain below)* __________________________________________________________________________________

__________________________________________________________________________________

__________________________________________________________________________________

__________________________________________________________________________________

__________________________________________________________________________________

__________________________________________________________________________________

__________________________________________________________________________________

__________________________________________________________________________________

12. Please indicate how much you agree or disagree with the following statements about research-level genetic testing:

**C. Questions about receiving the letter about your research-level genetic test results**

We are interested in your thoughts and feelings after receiving the letter about your research-level genetic test results, and how those results might have impacted your well-being.

***Directions:*** *Please indicate how much you felt each of the following after receiving the letter about your research-level genetic test results in the past two weeks.*

***Directions:*** *Please indicate how much you felt each of the following after receiving the letter about your research-level genetic test results in the past two weeks.*

12. Do you intend to share this information with your family members? __Yes __No

12a. Why or why not? *(Please elaborate on your answer to question 12 in the space provided below)*

____________________________________________________________________________________________________________________________________________________________

______________________________________________________________________________________________________________________________________________________________________________________________________________________________________________________________________________________________________________________________________________________________________________________________________

**D. Questions about your genetic counseling appointment**

We are interested in your thoughts on the genetic counseling appointment that you attended today regarding your research-level genetic testing results.

***Directions:*** *Please indicate how much you agree or disagree with the following statements about your genetic counseling appointment*

7. How did you feel about the timing of your genetic counseling appointment after receiving the letter about your research-level genetic test results in the mail? *(Please circle your answer)*

a. I would have preferred to be seen sooner

b. I thought the timing was fines

c. I could have waited longer to be seen

d. I have no preference on timing

**E. Questions about your decision to attend the genetic counseling appointment**

We are interested in your decision to come to the genetic counseling appointment to hear more about your research-level genetic testing result, and how you feel about that decision.

***Directions:*** *Please indicate how much you agree or disagree with the following statements about how you feel about your decision to hear about your research-level genetic testing result.*

6. Research-level genetic testing is not validated for clinical use, and therefore, it must be repeated through clinical genetic testing if the information is to be used to guide clinical care. How likely are you to complete confirmatory testing of the variant found during research testing?

a. Very likely

b. Somewhat likely

c. I’m not sure

d. Somewhat unlikely

e. Very unlikely

6a. Why or why not? *(please elaborate on your response to question 6)*

______________________________________________________________________________

______________________________________________________________________________

______________________________________________________________________________

______________________________________________________________________________

______________________________________________________________________________

______________________________________________________________________________

______________________________________________________________________________

****Note: The CHIP biobank is paying for your genetic counseling appointment as well as for the clinical genetic testing; please answer the following questions as if they were not paying for these services.***

7. How much would you have been willing to pay to have your result clinically validated?*

_________________ ($0-$3,000)

8. How much would you have been willing to pay for a genetic counseling?*

_________________ ($0-$500)

**F. Questions about genetics**

**Note: genome sequencing is genetic testing of all of someone’s genes.*

***Directions:*** *Please answer the following questions about how much you agree with the following statements about genome sequencing (genetic testing of all of someone’s genes).*

**H. Questions about your well-being**

***Directions:*** *Please answer the questions below by circling your answer.*

1. Over the **last two weeks**, how often have you been bothered by any of the following problems?

2. Over the **last two weeks**, how often have you been bothered by any of the following problems?

2a. If you checked off any problems, how difficult have these made it for you to do your work, take care of things at home, or get along with other people?

__Not difficult at all

__Somewhat difficult

__Very difficult

__Extremely difficult

**G. Participant background:**

1. What is the **highest** grade or level of school you have **completed** or the **highest degree** you have **received**? *(Please circle one answer only)*

a. Less than a high school graduate

b. High school graduate or GED

c. Some college

d. Associate’s degree

e. Bachelor’s degree

f. Master’s degree or professional degree (MA, MS, JD, etc.)

g. Doctoral degree (MD, PhD)

2. Do you work? __Yes __No

2a. If yes: __Full-time __Part-time

2b. If no: __Retired __On disability __Cannot find employment __By choice

3. Do you currently work in the sciences? __Yes __No __N/A

…in healthcare? __Yes __No __N/A

4. Do you have any (biological) children?

a. Yes, I have ____ children *(please write in the number of children you have)*

b. No, I do not have children

5. Do you have any full or half siblings?

a. Yes, I have ____ siblings *(please write in the number of siblings you have)*

b. No, I do not have siblings

6. When you first enrolled in the CHIP Biobank, did you know that your blood sample may be used by researchers for genetic testing?

a. Yes, I knew

b. No, I didn’t know

c. I do not remember

7. Have you had genetic testing previously? __Yes __No

7a. If yes, what were the results? *(If you don’t remember, please write so on the line)*

______________________________________________________________________________

______________________________________________________________________________

8. Have you ever been to see a genetic counselor before today? __Yes __No

8a. If yes, was it for your cardiac condition? __Yes __No

9. Do other family members (living or deceased) have the same cardiac condition as you? __Yes __No

9a. If so, how are they related to you? __________________________________________________

______________________________________________________________________________

______________________________________________________________________________

9b. Have any of these family members had genetic testing for the cardiac condition?

__Yes __No __I don’t know

9c. If yes, do you remember the results of the test? *(Please write results below)*

____________________________________________________________________________________________________________________________________________________________

***End of survey. Thank you for your participation!***

| Table S2. Demographic and Clinical Characteristics of Pathogenic Variant Carriers at the time of Dissection | | |
| --- | --- | --- |
| Variables | PV Carriers  N=26 | PV Disclosure  CLIA Confirmed  N=10 |
| Age of onset, years | 36 ± 15 | 44 ± 9 |
| Age of dissection, years | 39 ± 13 | 47 ± 8 |
| Male | 13 (50) | 3 (30) |
| Race (% white) | 22 (85) | 9 (90) |
| Ethnicity (% non-Hispanic) | 26 (100) | 10 (100) |
| Thoracic aortic indications |  |  |
| Root aneurysm | 14 (54) | 6 (60) |
| Ascending aneurysm | 12 (46) | 4 (40) |
| Arch aneurysm | 4 (15) | 2 (20) |
| Descending aneurysm | 5 (19) | 2 (20) |
| Max aneurysmal diameter, mm | 58 ± 14 | 53 ± 6 |
| Type A aortic dissection | 18 (69) | 6 (60) |
| Type B aortic dissection | 7 (27) | 4 (40) |
| Rupture | 1 (3.8) | 0 (0) |
| Risk Factors |  |  |
| HTN | 4 (15) | 3 (30) |
| Dyslipidemia | 2 (7.7) | 1 (10) |
| Smoking history (former/current) | 5 (19) | 2 (20) |
| Type 2 diabetes mellitus | 0 (0) | 0 (0) |
| Medications |  |  |
| ACE-I | 2 (7.7) | 2 (20) |
| Calcium channel blocker | 0 (0) | 0 (0) |
| ARB | 1 (3.8) | 1 (10) |
| Βeta-Blocker | 6 (23) | 3 (30) |

Values are median (IQR) or n (%).

Abbreviations: ACE-I=angiotensin converting enzyme inhibitor; ARB=Angiotensin II receptor blocker; CLIA: Clinical Laboratory Improvement Amendments; HTN=hypertension; pathogenic variant
